# BCOR abnormalities in endometrial stromal sarcoma

**DOI:** 10.1016/j.gore.2024.101672

**Published:** 2025-01-07

**Authors:** Abdulkareem Fayoumi

**Affiliations:** Department of Obstetrics and Gynaecology, Faculty of Medicine, King Abdulaziz University, Rabigh, Saudi Arabia

**Keywords:** Endometrial Cancer, Sarcoma, Grading, BCOR, Alteration, Fusions

## Abstract

Endometrial stromal tumors (ESTs) are uncommon mesenchymal tumors of the reproductive system associated with heterogeneous histomolecular features. According to the World Health Organization (WHO), ESTs are classified into benign endometrial stromal nodules (BESN) and endometrial stromal sarcomas (ESSs), which are further divided into low-grade and high-grade subtypes. High-grade ESS is frequently associated with YWHAE–NUTM2 gene fusions, while a newly recognized subtype with BCOR rearrangements, including fusions, alterations, and internal tandem duplications (ITDs), has recently been incorporated into the molecular classification of ESS. BCOR, a transcriptional corepressor of BCL-6, contributes to tumor progression through its role in polycomb repressive complex 1 (PRC1), underscoring its importance in oncogenesis and potential as a therapeutic target. Advances in molecular diagnostics, such as next-generation sequencing (NGS) and fluorescence in situ hybridization (FISH), have improved the precision of diagnosing BCOR-altered ESS, enabling better prognostic stratification. These findings also support the development of targeted therapies, including CDK4/6 inhibitors and immunotherapies targeting PD-1 and CTLA-4 pathways. Despite these advancements, barriers such as limited access to molecular diagnostics and the high cost of novel therapies remain significant challenges. This review bridges molecular and clinical insights into ESS, emphasizing the diagnostic, prognostic, and therapeutic implications of BCOR rearrangements. By integrating these advances into clinical practice, it aims to improve outcomes for patients with this rare and aggressive malignancy.

## Highlights

• *BCOR* abnormalities play a crucial role in high-grade endometrial stromal sarcomas.
• The most common subset is *ZC3H7B-BCOR* gene fusion,
• *BCOR* abnormalities are commonly detected through IHC or molecular testing,
• Current treatments include surgery, with possible radiotherapy and chemotherapy.
• Emerging therapies targeting the tumor microenvironment or epigenetic dysregulation are being explored for improved outcomes.

## 1 Introduction

**Fig. 1:**
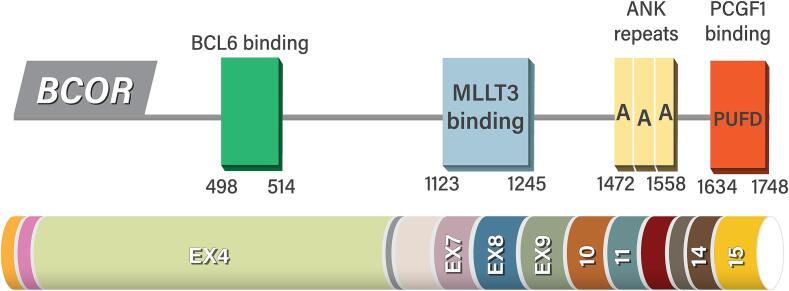
Structure and functional domains of *BCOR*, featuring BCL-6 and MLLT3 binding regions, ANK repeats, and the PUFD domain. Additionally, a schematic representation of the exon arrangement is displayed below the protein domains.

Epigenetic regulation is a mechanism that influences the epigenetic state of a cell, without changing the DNA sequence. It involves modifications to the genome that control gene expression, affecting how genes are turned “on” or “off.” Epigenetic regulators are potential proto-oncogenes or tumor suppressors that can influence cancer development or regression depending on the changes that occur (Dawson and Kouzarides, 2012 Jul 6). *BCOR* is a common epigenetic regulator specifically important in cell differentiation and development and its role is significant because its alterations, mutations, fusions, or deletion can lead to abnormal gene expression, contributing to the development of cancers. BCOR is a transcriptional corepressor of BCL-6 that amplifies its transcriptional repression activity and is situated at the Xp11.4 locus on the X chromosome (Huynh et al., 2000) [Fig. 1]. The gene is encoded by 14 exons that lead to 1700 amino acids (Huynh et al., 2000)**.** *BCOR* is mediated by the BCL-6 and *PUFD* binding domains, which interact with BCL-6, playing a role in cell differentiation and modification, and participating in the Polycomb Repressive Complex 1 (PRC1) (Astolfi et al., 2019 May). Polycomb Repressive Complexes (PRC1 and PRC2) are molecular assemblies that participate in histone modification, particularly by adding methyl groups to the H3K27 site on histone H3 (Dawson and Kouzarides, 2012 Jul 6, Huynh et al., 2000, Astolfi et al., 2019 May, Blackledge et al., 2015). The core of the PRC2 complex associates with various proteins to regulate its enzymatic activity, while PRC1 contains a core of the *RING1* protein. PRC1 complexes are categorized into two forms of canonical and noncanonical variant (Blackledge et al., 2015). The canonical variants bind to *H3K27me3* and is implemented by the PRC2 complex (Schuettengruber et al., 2017). In contrast, the noncanonical PRC1 complex binds to *RYBP* and *YAF2*, interacting with other proteins to form the PRC1 complex. Despite the complexity of these mechanisms, BCOR contributes to the formation of noncanonical PRC1 variants (van den Boom et al., 2016). In the PRC1.1 complex, *BCOR* interacts with *PCGF1* through its *PCGF* Ub-like fold discriminator domain. PRC1.1 represses gene expression by ubiquitinating Lys119 on histone H2A (*H2AK119*). Germline mutations that disrupt the function of genes encoding PRC proteins have been associated with developmental abnormalities and a range of human cancers (Van der Lugt et al., 1994, Akasaka et al., 1996). *BCOR* mutations are directly associated with cancer development by altering the protein's RNA recognition through alternative splicing at the pre-mRNA level (Lee et al., 2015). The role of *BCOR* ocogeneic alteration were found in clear cell sarcoma of the kidney [CSSK], a pediatric renal sarcoma and other sarcomas such as primitive myxoid tumor of the infancy, rhabdomyosarcoma as well as primary central nervous system (CNS) neuroectodermal tumor, medulloblastoma, retinoblastoma, myelolymphoid neoplasms, and salivary gland cancers (Lee et al., 2015, Gooskens et al., 2012, Argani et al., 2000, Boo et al., 2009, Mirkovic et al., 2015, Knutson et al., 2013, Bantignies and Cavalli, 2011, Marin ~o-Enriquez et al., 2018, Hoffman et al., 2016, Argani et al., 2017, Haferlach et al., 2014, Moreira et al., 2015). *BCOR* alteration was also identified in gynecological malignancy (Panagopoulos et al., 2013, Cramer et al., 2017, Morris et al., 2017, Petrini et al., 2013). While comprehensive studies on the role of *BCOR* mutations in carcinomas are still scarce, it is evident that *BCOR* abnormalities frequently occur in uterine endometrial carcinoma (Morris et al., 2017, Petrini et al., 2013, Garc ´ıa-Sanz et al., 2017). While *BCOR* alterations are commonly observed in endometrial stromal sarcoma (ESS), they have also been identified in a subset of ossifying fibromyxoid tumors (Antonescu et al., 2014 Feb).

## 2 Endometrial stromal sarcoma [ESS]

Endometrial stromal sarcomas [ESSs] are rare and complex mesenchymal uterine neoplasms with heterogeneous morphological, histochemical and genetic features. They constitute 10–15 % of uterine mesenchymal tumors (Silverberg and Kurman, 1992). The annual incidence of ESS is about 0.3 per 100,000 women (Abeler et al., 1970). Around 50 % of ESSs are diagnosed in premenopausal women, with the majority being identified at stage I (Felix et al., 2013). Histologically, they resemble endometrial stromal cells during the proliferative phase of the menstrual cycle (Norris and Taylor, 1966). ESS is characterized as a malignant, infiltrative neoplasm with a mitotic rate of more than 10 figures per high-power field (HPF). Patients with ESS have a five-year survival rate of approximately 55 % (Lee et al., 2015). Recently, the WHO revised the classification of ESS based on molecular profiling, dividing it into low-grade, high-grade, and undifferentiated uterine sarcomas (USS). These subtypes differ in both histological features and molecular characteristics. The 2020 WHO subclassification has significantly enhanced the accuracy of diagnosis and prognosis for ESS. Clinically, low-grade ESS is the most common type, alongside benign endometrial stromal nodule [BESN] and high-grade ESS (Gadducci et al., 2023). One third of low-grade ESSs do not usually have interacted fusions compared to high-grade ESSs (Gadducci et al., 2023).

According to WHO, low-grade ESSs usually carry *JAZF1-SUZ12* fusion while high-grade ESSs usually carry *YWHAE–NUTM2* fusion [translocation t(10;17) (q22;p13)] that are often associated with low-grade morphology (Lee et al., 2015). Some low-grade ESS cases have been reported to harbor the *YWHAE-NUTM2* fusion, a molecular feature typically associated with high-grade ESS (Brahmi et al., 2020). Other gene mutations identified in low-grade ESSs have been rarely reported in the literature such as *JAZF1-PHF and JAZF1-BCORL1* (Micci et al., 2006, Allen et al., 2017).

## 3 Endometrial stromal sarcoma [ESS] and BCOR abnormalities

In the past decade, besides the *YWHAE-NUTM2* fusion, several new molecular subtypes have been identified in high-grade ESSs compared to low-grade ESSs. *BCOR* gene abnormalities, including mutations, fusions, alterations, or duplications, have emerged as newly recognized genetic aberrations in ESSs (Gadducci et al., 2023) **[**Table 1].

**Table 1:** Published studies involving cases of all subtypes of Endometrial Stromal Sarcoma [ESS] with *BCOR* abnormalities**.**

**ESS type**	**Gene involved**	**Reference**
**Low-grade ESS**	BCORL1 [EPC1-PHF1] BCOR [JAZF1-SUZ12]	(Micci et al., 2016, Dickson et al., 2018)
	BCOR [EPC1-BCOR]	(Dickson et al., 2018)
**High-grade ESSs**	BCOR [alteration]	(Brahmi et al., 2020)
	ZC3H7B-BCOR	(Panagopoulos et al., 2013, Antonescu et al., 2014 Feb, Hoang et al., 2017, Micci et al., 2016, Lewis et al., 2018, Chiang et al., 2017, Juckett et al., 2019, Lin et al., 2020, Kommoss et al., 2020, Zhi et al., 2022, Zhao et al., 2023, Mansor et al., 2019, Ondič et al., 2020, Ling et al., 2022, Wagh et al., 2023)
	BCOR ITD	(Juckett et al., 2019)
**UUSs**	BCOR [HMGA2-RAD51B]	(Momeni Boroujeni et al., 2020)

High-grade ESS frequently involves *BCOR* as part of a fusion gene with *ZC3H7B* [*ZC3H7B-BCOR*; translocation t(X;22) (p11; q13)] (Panagopoulos et al., 2013, Hoang et al., 2017, Micci et al., 2016, Lewis et al., 2018, Chiang et al., 2017, Juckett et al., 2019, Lin et al., 2020). *ZC3H7B–BCOR* ESS was also identified independently in a subgroup of low-grade ESS (Hoang et al., 2017). Lin *et al*. studied a large series of 40 cases with ESS contained *BCOR* rearrangement and *BCOR* Internal Tandem Duplication (ITD) (Lin et al., 2020). They found 31 cases with canonical *ZC3H7B-BCOR* fusion as well as 8 cases with novel *BCOR* gene rearrangement partners. ITDs usually involves exon 15 of *BCOR*. Undifferentiated uterine sarcoma [UUS] is another type of ESS neoplasms that never had any *BCOR* alteration reported. However, the tumor expresses p16, *BCOR*, Cyclin D1, CD10, estrogen receptor [ER] and progesterone [PR] (Kurihara et al., 2010). Thus, some UUSs could be misdiagnosed as high-grade ESSs (Cotzia et al., 2019). A single case harboring the *HMGA2-RAD51B* fusion has also been reported, showing high expression of *NTRK3, FGFR3, RET, BCOR, GLI1,* and* PTCH1* (Momeni Boroujeni et al., 2020). UUSs with *BCOR*-ITD exhibits uniform nuclear features (Akaev et al., 2021;11[3]:429., Kim et al., 2022).

## 4 Histological features of ESS including ESS with BCOR abnormalities

The histological differences between BESN and ESS are quite challenging because *BCOR* alteration or other common mutations are not characteristically found in BESN (Kurman et al., 2014). In benign nodules, there is no invasion deeper than 3 mm, no lymphovascular invasion (LVI), and mitotic figures are fewer than 10 per high-power field (HPF). BESN shows minimal expression of CD10, ER, and is focally positive for the PR (Nomura et al., 2020). They typically don’t express p53 protein and Ki-67 is usually very low (Nomura et al., 2020). The most frequent features distinguish BESN from ESS are mitosis and surrounding tissue invasion. Myometrial invasion is a common histological finding differentiating benign nodules from low-grade ESS and can be clearly highlighted with muscle stains (Nomura et al., 2020). Low-grade ESS is characterized by more than 10 mitotic figures per HPF and the presence of LVI (Makise et al., 2019). While CD10 is not a specific or sensitive marker for distinguishing between benign lesions and low-grade ESS, ER and PR show strong expression in low-grade ESS (Alan et al., 2019, Park et al., 2018). Additionally, strong androgen receptor (AR) expression has been observed, with weak positivity reported in 95 % of low-grade ESS cases (Park et al., 2018, Yadav et al., 2019). In contrast, high-grade ESSs usually show negative ER and PR expressions with minimal expression of CD10 (Cotzia et al., 2019). These histochemical distinctions should not be made solely based on a biopsy; the diagnosis should be confirmed after tumor resection. Furthermore, these features are not sufficient to differentiate between the molecular subtypes of all ESSs.

The histological difference between different molecular subtypes of high-grade ESSs are not usually specific. High-grade ESSs with *YWHAE-NUTM2* fusion may be characterized by high-grade round and low-grade fibromyxoid spindle cell components and they are commonly reactive to cyclin D1 and *BCOR* antibodies (Lee et al., 2015, Cotzia et al., 2019, Kim et al., 2022). Compared to patients with low-grade ESS, those with *YWHAE-NUTM2* high-grade ESS experience earlier recurrence and have a higher likelihood of succumbing to the disease (Lee et al., 2015, Sciallis et al., 2014). High-grade ESS with *BCOR* rearrangements, such as *ZC3H7B–BCOR* ESS, frequently exhibit neoplastic or infarct-type necrosis (Lewis et al., 2018). In some cases, moderate to severe cytological atypia is observed, marked by condensed chromatin, prominent nucleoli, and enlarged nuclei, frequently accompanied by an epithelioid or small round cell component (Kim et al., 2022). LVI is a common finding, and both ER and PR expressions are variable. Recently, Pan-Trk expression has been observed in some cases (Lin et al., 2020, Solomon et al., 2020). Other *BCOR* gene rearrangements have also been documented in the literature, with some displaying closely similar histological patterns. Recent studies have reported amplification of *MDM2, FRS2, and CDK4*, along with the loss of *CDKN2A* in certain cases (Brahmi et al., 2020, Lin et al., 2020, Kommoss et al., 2020, Thiryayi et al., 2022). Kommoss *et al*. identified amplification of the 12q15 region involving the *MDM2* locus in 5 cases of high-grade ESS with *BCOR* rearrangement (Kommoss et al., 2020). Those cases showed brisk mitosis, severe nuclear atypia and variable necrosis. At least focal myopermeative growth was identified in all cases, with focal myxoid stromal changes. A comprehensive genomic profiling performed by Lin *et al* revealed high frequency of *CDK4* and *MDM2* amplification in about 40 % of *BCOR*-rearranged cases (Lin et al., 2020). Research into therapeutically targeting the *CDK4* pathway in a subset of ESS patients shows promise, indicating that alterations in this pathway, for which targeted therapies are available, may contribute to the pathogenesis of *BCOR*-rearranged ESS. Mariño-Enriquez *et al*. identified three cases of ITD-*BCOR*, characterized by the typical immunophenotypic profile of ITD-*BCOR*-positive tumors, including overexpression of cyclin D1 and *BCOR*, along with negative expression for ER and PR (Marin ~o-Enriquez et al., 2018, Akaev et al., 2021;11[3]:429.).

The transformation of ESS from low-grade to high-grade was also reported (Zou et al., 2020). However, this transformation was detected in cases where *BCOR* alterations is absent. Zou *et al* screened 12 cases with low-grade type transformed into high-grade within 10 years (Zou et al., 2020). They found that high-grade transformation only occurs with *JAZF1* and *PHF1* rearrangements few years after initial diagnosis.

## 5 BCOR rearrangement identification in ESS

Altered *BCOR* expression can be assessed using immunohistochemistry (IHC) and genetic testing. While IHC is typically specific, it lacks sensitivity for detecting *BCOR* alterations, as it can identify the presence of a rearrangement but not the specific type of genetic mutation. The anti-*BCOR* antibody (clone C-10, Santa Cruz, Leica, Germany) is commonly used, though proper antibody validation is essential for accurate, standardized results (Lin et al., 2020). Fluorescence in situ hybridization (FISH) is a practical genetic testing method. FISH can be performed on 5-μm formalin-fixed, paraffin-embedded tumor sections using probes flanking the *YWHAE, BCOR, and ZC3H7B* genes (Lewis et al., 2018). Custom probes can be created from bacterial artificial chromosome (BAC) clones harboring *YWHAE* (RP11-105D11, RP11-1142D6), *BCOR* (RP11-21D3), and *ZC3H7B* (RP11-1078O11) genes, obtained from BAC/PAC Resources (Oakland, CA, USA) (Antonescu et al., 2014 Feb). Gene rearrangement is confirmed when break-apart signals appear in more than 20 % of nuclei. Next-generation sequencing (NGS) is another method for detecting *BCOR* rearrangements, utilizing RNA sequencing to identify gene fusions and oncogenic isoforms.

## 6 Clinical implications of BCOR abnormalities in endometrial stromal sarcomas

**Fig. 2:**
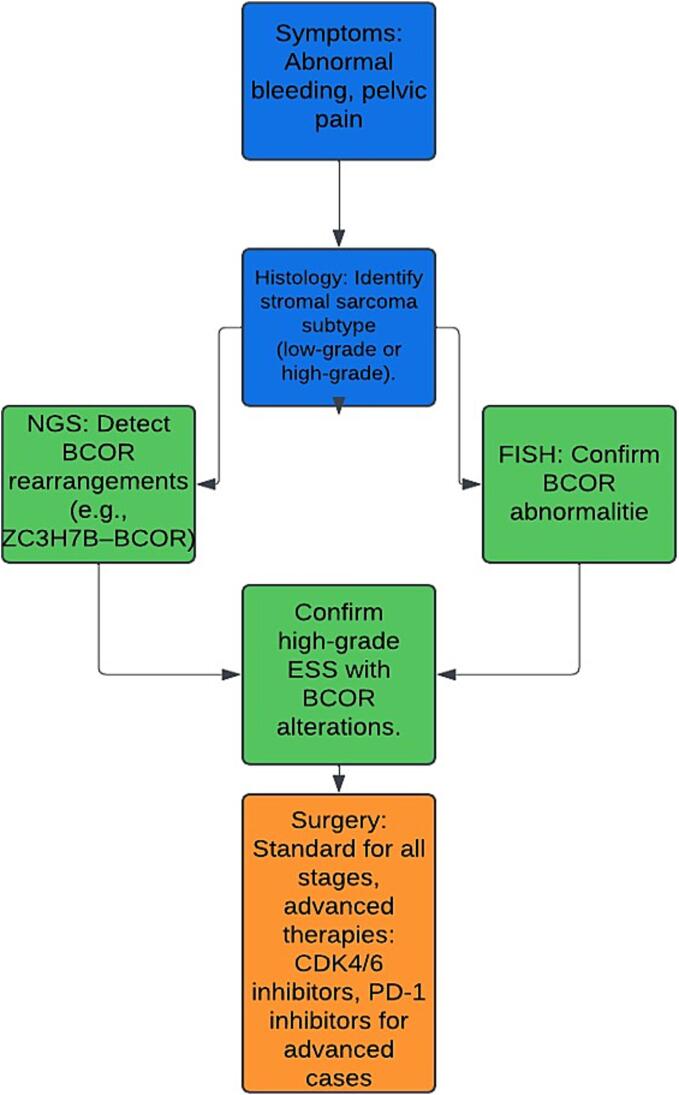
Diagnostic workflow for BCOR-altered ESS, illustrating the integration of clinical and molecular diagnostic tools into patient management. High-Grade ESS: Decision-Making Algorithm for BCOR-Altered and Non-BCOR Cases.

The identification of BCOR abnormalities in high-grade endometrial stromal sarcomas (ESSs) has transformed the diagnostic and prognostic landscape of this rare tumor. Molecular diagnostics, such as next-generation sequencing (NGS) and fluorescence in situ hybridization (FISH), play a pivotal role in detecting BCOR rearrangements, including ZC3H7B–BCOR fusions and internal tandem duplications (ITDs) (Panagopoulos et al., 2013, Lewis et al., 2018, Zhi et al., 2022) [Fig. 2). The integration of these advanced techniques into routine diagnostic workflows has significantly improved the accuracy of ESS subtyping, ensuring appropriate treatment stratification (Micci et al., 2016, Chiang et al., 2017, Cotzia et al., 2019).

From a prognostic perspective, BCOR-altered ESSs exhibit distinct clinical behavior characterized by increased aggressiveness and poorer outcomes compared to other subtypes (Chiang et al., 2017, Lewis et al., 2018, Zhi et al., 2022). This emphasizes the need for early and precise identification to guide therapeutic decision-making. For instance, while low-grade ESSs respond well to hormonal therapies, high-grade ESSs with BCOR rearrangements may benefit from novel targeted treatments currently under investigation (Gadducci et al., 2023, Mansor et al., 2019).

In clinical practice, the presence of BCOR rearrangements can influence management decisions by identifying patients who are candidates for advanced therapies. Targeted therapeutic approaches, such as CDK4/6 inhibitors and immunotherapies like PD-1 inhibitors, leverage the molecular vulnerabilities of BCOR-altered ESS (Kim et al., 2022, Kommoss et al., 2020). Furthermore, identifying BCOR abnormalities aids in selecting patients for clinical trials, driving the development of personalized treatments for this aggressive subtype (Juckett et al., 2019, Lin et al., 2020, Dickson et al., 2018).

However, practical challenges remain, including the accessibility and cost of molecular diagnostic tools in resource-limited settings (Capozzi et al., 2020). To address these barriers, it is critical to develop cost-effective diagnostic strategies and foster collaborations between academic institutions and healthcare systems. By integrating BCOR findings into clinical guidelines and enhancing molecular diagnostic capacity, the management of BCOR-altered ESSs can shift toward a more precise and patient-centric approach (Mansor et al., 2019, Ondič et al., 2020).

## 7 Current therapeutic Strategy of ESS and ESS with BCOR rearrangements

**Fig. 3:**
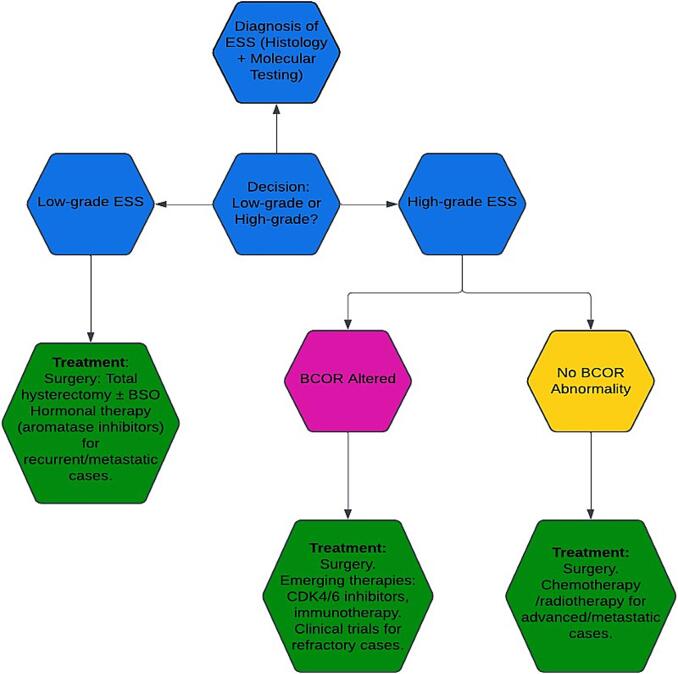
Treatment algorithm for high-grade ESS, distinguishing between BCOR-altered and non-BCOR cases. The diagram outlines the diagnostic pathway from histological and molecular testing to therapy selection, including surgery, targeted therapies for BCOR-altered.

The standard treatment for ESS generally involves surgical intervention, typically including a total hysterectomy and bilateral salpingo-oophorectomy (BSO), without morcellation, especially in early-stage cases of both low-grade and high-grade ESS (Gadducci et al., 2023). The treatment algorithm for high-grade ESS, distinguishing between BCOR-altered and non-BCOR cases, is summarized in Fig. 3. For lesions limited in the uterus, en bloc removal of the affected area may be suggested. The option of ovarian preservation should be discussed in young women with stage I disease, in case fertility is needed. For patients with positive lymph nodes, pelvic nodal dissection and revision do not significantly impact clinical outcomes. The five-year overall survival (OS) for most early-stage cases is approximately 97 % following surgery (Gadducci et al., 2023). While controversial, the use of radiotherapy (RT) and hormonal therapy has not been shown to improve OS in early-stage patients. However, the NCCN guidelines recommend aromatase inhibitors as the first-line treatment for low-grade ESS (Network and Neoplasms, 2021). In advanced high-grade ESS, surgical resection of all visible disease followed by hormonal therapy is advised (Gadducci et al., 2023). Cytoreduction by removing the metastatic deposit is the standard approach for metastatic high-grade ESS (Capozzi et al., 2020).

The standard treatment approaches described for ESS remain the same, even when *BCOR* gene abnormalities are detected. Due to the ongoing debate over the effectiveness of radiotherapy (RT) and chemotherapy (CT), new strategies targeting the tumor immune microenvironment through systemic immunotherapy have recently been explored as potential alternatives (Rizzo et al., 2019). The connection between the immune system and cancer regulation was first noted in 1866 when German patients with sarcoma developed erysipelas infections (Roberts et al., 2015). Over decades, immune checkpoint inhibitors include programmed cell death protein-1 [*PD1*] and CTL-associated antigen 4 [*CTLA-4*] were initiated. Although *PD-L1* expression in ESS has not been fairly explored, *PD-L1* was expressed in more than 75 % of uterine cancer patients with primary disease (Engerud et al., 2020). This suggests that *PD-1* could play a significant role in the treatment of ESS. Nivolumab, a PD-1 inhibitor, is currently being tested in clinical trials, both alone and in combination with Ipilimumab, which targets *CTLA-4* (ClinicalTrials.gov., 2022). More upcoming strategies focus on targeted and immunotherapeutic approaches. CDK4/6 inhibitors in cases with altered *CDK4* and *BCOR* are also under investigations. CDK4/6 inhibitors are a class of targeted therapies that block the activity of cyclin-dependent kinases 4 and 6 [CDK4/6], which are proteins involved in regulating cell cycle progression. CDK4/6 inhibitors, such as palbociclib, ribociclib, and abemaciclib, work by halting cancer cell division, potentially slowing tumor progression. Clinical trials are currently exploring their efficacy in *BCOR*-altered sarcomas as part of personalized cancer treatment strategies (Lin et al., 2020).

## 8 Conclusion

BCOR abnormalities are pivotal in the pathogenesis of high-grade endometrial stromal sarcomas (ESS), particularly in the ZC3H7B-BCOR gene fusion subtype, which is associated with aggressive clinical behavior, high recurrence rates, and poor outcomes. Histologically, these tumors exhibit high-grade spindle morphology, brisk mitotic activity, necrosis, and negative hormonal receptors, distinguishing them from low-grade ESS. Advances in molecular diagnostics, such as immunohistochemistry (IHC), fluorescence in situ hybridization (FISH), and next-generation sequencing (NGS), have greatly enhanced the accuracy of diagnosing BCOR-altered ESS, improving prognostic stratification and guiding treatment decisions.

Despite the progress in diagnostics, therapeutic strategies for BCOR-altered ESS remain limited, with surgery often being the cornerstone of treatment, supplemented by radiotherapy and chemotherapy. Emerging therapies targeting the tumor microenvironment and epigenetic dysregulation, including immunotherapies and CDK4/6 inhibitors, hold promise for improving outcomes in this aggressive subtype. Future multi-institutional studies are essential to refine treatment protocols and integrate novel therapies into clinical guidelines. By bridging molecular insights with clinical practice, this review highlights the need for a more personalized and effective approach to managing BCOR-altered ESS.

## CRediT authorship contribution statement

**Abdulkareem Fayoumi:** Writing – review & editing, Writing – original draft, Methodology, Formal analysis, Conceptualization.

## Declaration of Competing Interest

The authors declare that they have no known competing financial interests or personal relationships that could have appeared to influence the work reported in this paper.
